# Pathway and gene-set activation measurement from mRNA expression data: the tissue distribution of human pathways

**DOI:** 10.1186/gb-2006-7-10-r93

**Published:** 2006-10-17

**Authors:** David M Levine, David R Haynor, John C Castle, Sergey B Stepaniants, Matteo Pellegrini, Mao Mao, Jason M Johnson

**Affiliations:** 1 Rosetta Inpharmatics LLC, a wholly owned subsidiary of Merck and Co., Inc., Terry Avenue North, Seattle, WA 98109, USA; 2 Department of Radiology, University of Washington, Seattle, WA 98195, USA; 3 Department of MCD Biology, University of California at Los Angeles, Los Angeles, CA 90095, USA

## Abstract

**Background:**

Interpretation of lists of genes or proteins with altered expression is a critical and time-consuming part of microarray and proteomics research, but relatively little attention has been paid to methods for extracting biological meaning from these output lists. One powerful approach is to examine the expression of predefined biological pathways and gene sets, such as metabolic and signaling pathways and macromolecular complexes. Although many methods for measuring pathway expression have been proposed, a systematic analysis of the performance of multiple methods over multiple independent data sets has not previously been reported.

**Results:**

Five different measures of pathway expression were compared in an analysis of nine publicly available mRNA expression data sets. The relative sensitivity of the metrics varied greatly across data sets, and the biological pathways identified for each data set are also dependent on the choice of pathway activation metric. In addition, we show that removing incoherent pathways prior to analysis improves specificity. Finally, we create and analyze a public map of pathway expression in human tissues by gene-set analysis of a large compendium of human expression data.

**Conclusion:**

We show that both the detection sensitivity and identity of pathways significantly perturbed in a microarray experiment are highly dependent on the analysis methods used and how incoherent pathways are treated. Analysts should thus consider using multiple approaches to test the robustness of their biological interpretations. We also provide a comprehensive picture of the tissue distribution of human gene pathways and a useful public archive of human pathway expression data.

## Abstract 2

A comparison of five different measures of pathway expression and a public map of pathway expression in human tissues are presented.

## Background

Microarray experiments typically measure mRNA populations in tissue samples and changes in those populations following perturbations. The main result of a microarray experiment is a list of genes whose expression is significantly changed relative to a comparison sample. This gene list will typically contain hundreds to thousands of genes, and biological interpretation of this list is often the most time-consuming analysis step. To interpret the set of differentially regulated genes, a scientist may order them by statistical significance or expression fold-change and then work through the list, picking out familiar genes, grouping genes that appear to have similar functions, and conducting literature searches to help understand the functions of unfamiliar genes. Eventually, most of the genes in the list are grouped and understood in terms of biological processes that have meaning to the scientist, such as the activation or repression of particular pathways or sets of genes with common function. Recent increases in available gene annotation and pathway databases have made it possible and worthwhile to complement this manual approach with automated analysis of pathway expression changes, the coordinated induction or repression of multiple genes in a predefined pathway, by reference to a database of known pathways. Here, we present and examine approaches that pre-filter gene sets in a database for correlated behavior over multiple experiments and then test the differential regulation of each gene set or pathway. In what follows, we use the terms 'pathway' and 'gene set' interchangeably.

The idea of inspecting output gene lists from microarray experiments for statistical enrichment of previously annotated gene sets emerged with early microarray studies [1,2]. Over time the approach has become more systematic, relying on the use of keyword databases such as Swiss-Prot [3], MEDLINE [4], and Gene Ontology [5-12] as annotation sources. Several tools have also been developed to help facilitate automation of enrichment analyses from a gene list, generally using Gene Ontology categories [6,9,13-15]. Recently, there has been a trend to look for enrichment not just in the analysis of individual experiments, but among different classes of experiments [16] and in larger compendia of expression data, including a set of 55 mouse tissues [17], a database of expression from 19 human organs [18], and a meta-analysis of 22 human tumor types [19]. Many different methods for measuring pathway expression have been used, but to date no substantial systematic comparison of multiple methods over multiple independent data sets has been performed.

Here, we compare five different methods for defining pathway expression over nine publicly available mRNA expression data sets. Many pathways are identified by all methods as significantly changed. However, there are also a number of pathways that are only identified as significantly changed by a subset of the measures. These results are dependent on whether and to what extent pathways with incoherent (uncorrelated) expression [20] are removed. Biological interpretation of the results may thus be dependent upon the choice of pathway expression metric and how incoherent pathways are handled. Following the comparison of methods, we apply these methods and use coherence filtering to construct a public reference map of human pathway expression data. This map is a two-dimensional matrix of 290 pathways by 52 samples, showing which pathways are upregulated or downregulated in each of these normal tissues and cancer cell lines. A high-resolution version of this map and all expression data are freely available [21]. The resulting map of the expression of human pathways and other gene sets is consistent with the known tissue specificities of many molecular processes and suggests new insights into the action of different pathways in human tissues. Finally, we demonstrate the use of pathway measurements to refine and correct errors in pathway annotations.

## Results and discussion

### Measuring gene set expression

**Figure 1:**
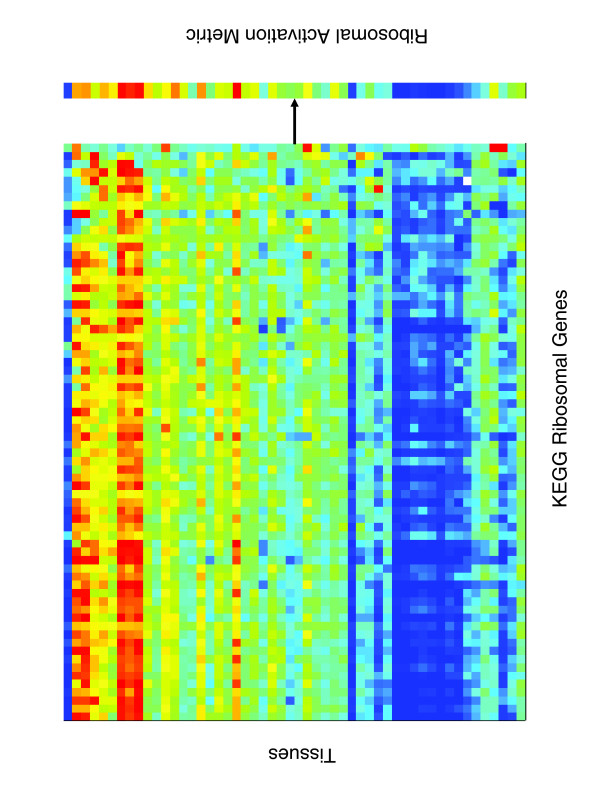
Example of pathway activation calculation. Shown on the left are the expression levels of the 70 genes in the KEGG Ribosome gene set measured across a set of tissue samples. The columns are genes and the rows are tissues. Bright red indicates overexpression of a gene relative to a pool of all tissues, and dark blue significant underexpression. For each tissue, the pathway activation metric (represented by the black arrow) is used to calculate a corresponding scalar value that captures the predominant expression of the genes in the Ribosome gene set in that tissue. Taken together, these scalar values constitute the pathway activation metric vector shown on the right.

We compared the following five pathway 'activation metrics' for mapping the vector of expression values for all genes in a pathway to a scalar value representing the expression level of the pathway (Figure 1).

#### *Z*-score

Suggested recently in a microarray context [22,23], the *Z *score used here represents the difference (in standard deviations) between the error-weighted mean of the expression values of the genes in a pathway and the error-weighted mean of all genes in a sample after normalization. The result reflects both the magnitude and relative direction of a gene set's expression.

#### Hypergeometric

This metric measures the enrichment of transcriptionally active genes in a gene set by calculating a *p *value using the hypergeometric distribution. It requires the user to define a statistical threshold for significant induction or repression. To reflect directionality, induced and repressed genes are considered separately and the more significant of the two *p* values, along with the appropriate sign (negative if repressed genes were more significant, positive otherwise) is used.

#### Principal component analysis

The first principal component of the expression values in a gene set captures the dominant linear mode of covariation of the expression of the genes in that gene set.

#### Wilcoxon *Z*-score

This metric is the mean rank of the genes in the pathway (among all genes on the microarray), normalized to mean zero and standard deviation one.

#### Kolmogorov-Smirnov

The Kolmogorov-Smirnov (KS) statistic used here represents the maximum absolute deviation between the cumulative distribution function (CDF) of the expression values of the genes in the pathway and the CDF of all the genes in the experiment. To reflect directionality, we give a sign to the KS statistic according to whether the maximum absolute deviation arose from a positive or negative difference between the two CDFs.

### Coherence of gene set expression

Separately, we developed a simple metric to quantify the degree of co-regulation, or 'coherence', of the genes in the gene set over a given set of experimental samples. Pathways whose component genes are perturbed in a correlated manner are, in general, more likely to be relevant to biological interpretation of the experimental results, while pathways whose component genes demonstrate uncorrelated, incoherent expression are less likely to be relevant to the biological meaning of the list of perturbed genes. We also explored the hypothesis that different metrics of gene set activation are more likely to give concordant results for coherent pathways than for incoherent pathways. As the measure of coherence, we use the percentage of total variance of the expression values within the gene set captured by the first principal component across all samples. Unlike some of the other possible methods for measuring coherence, this measure is not biased against gene sets whose component genes are regulated in opposing directions over the samples, as long as the relative behavior of pairs of component genes is consistent across the samples. This is not a perfect filter, however, since it may miss certain activated pathways, for example, certain signaling pathways that may not exhibit a strong transcriptional response.

To test the ability of each metric to convert gene-level expression into pathway expression, we assembled a database of 1,401 annotated human pathways and gene sets: 120 from KEGG [24,25], 1040 from the Biological Process hierarchy of the Gene Ontology (GO) database [26], and 241 from the Cellular Component hierarchy of GO. For evaluation of the five pathway metrics described above we selected nine recent data sets from the GEO database [27] (GDS1062 [28], GDS1067 [29], GDS1210 [30], GDS1220, GDS1221 [31], GDS1231 [32], GDS1332 [33] and two data sets from GDS1239 [34]). Each data set contains two subsets of samples: a baseline set of samples and one subset of samples representing a disease state or a different disease state from the baseline (Table 1). Since each of the two subsets contains multiple relatively similar samples, these are used as biological replicates to estimate the false discovery rate (FDR) for each pathway activation metric in the analysis below. Although this is not the same as comparing two sets of control and experimental samples each consisting of replicates of the same tissue from genetically identical animals, it is representative of comparisons made in the literature using clinical samples from different patients, and diversity of samples within each subgroup increases the likelihood that the differentially regulated pathways will generalize to other samples of the same types. In addition, the performance of each activation metric, although variable in absolute terms across datasets, remained consistent relative to other metrics across datasets, which increases our confidence that the differences described below are real.

**Figure 2:**
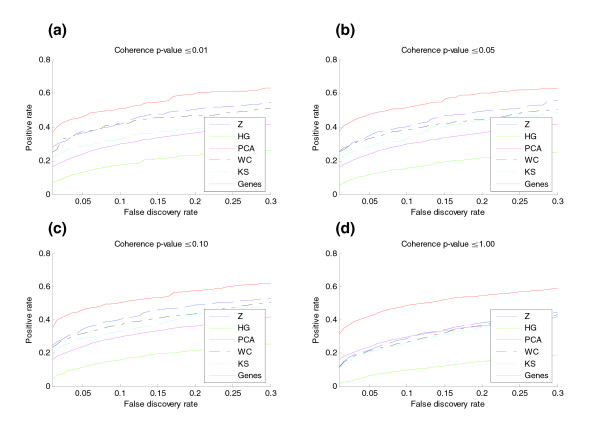
ROC analysis was used to compare the detection sensitivity of five metrics of gene set activation and individual genes to discriminate between two different subgroups in nine different data sets (Table 1). A Wilcoxon rank sum test was used to test the null hypothesis for each gene set and individual gene that the two different subgroups groups were drawn from the same distribution. **(a-d) **The four graphs show results using four different *p *value thresholds for pathway coherence. Shown on the y-axis is the positive rate: the percentage of the gene sets or genes declared different between the two subgroups as a function of the FDR (the x-axis). The results are averaged over all nine data sets. The operating range of the X axis, [0.0, 0.3] was chosen to correspond to the range of FDRs that might be acceptable in practice. ROC curves were also calculated for each of the nine data sets individually (Supplemental Figures F1 to F9 in Additional data file 1). HG, hypergeometric; WC, Wilcoxon *Z *score; *Z*, *Z *score.

Using receiver-operator characteristic (ROC) curves we measured the sensitivity of each pathway activation metric to differences between the two sample subsets in each of the nine independent data sets as a function of FDR. For comparison, we also measured the sensitivity of the expression vectors of individual genes (see Materials and methods). To test the hypothesis that coherence-filtering would affect the results, we studied each metric for its performance on gene sets of varying coherence (coherence *p *values ≤ 0.01, 0.05, 0.10, and 1.0). Sensitivities at a given FDR were averaged over all nine data sets for each of the metrics and for each coherence threshold. The combined performance results are shown in Figure 2. Results for the individual data sets are provided as Supplemental Figures F1 to F9 in Additional data file 1 . Using coherent gene sets, all activation metrics except the hypergeometric were more sensitive in detecting differences between the two replicate groups than was a comparison using the expression of individual genes. The observation that small but coordinated changes in expression may be easier to detect at the pathway level than at the gene level has been noted previously [16]. Qualitatively, this can also be observed in Figure 1, in which the expression of the individual genes is somewhat noisy, but the pathway activation metric captures the predominant signal more clearly.

The relative performance of the different metrics varied widely over the data sets (see Supplemental Tables T1 to T9 in Additional file 2 and Supplemental Figures F1 to F9 in Additional data file 1). The best performing metric also varied over the data sets; each of *Z *score, KS, Wilcoxon *Z *score, and principal component analysis (PCA) was the most sensitive for at least one of the data sets. In general, for data sets with very different samples and thus large numbers of genes with significant differential expression ('signature genes'), all of the metrics tended to perform well and the choice of metric is less critical. However, for data sets with lower numbers of signature genes, results were much more variable. For example, because the hypergeometric metric considers only the set of predefined signature genes, it performed poorly when there were very few such genes and should not be used in such circumstances. The other metrics take into account the expression of each gene in a pathway, regardless of whether the individual gene expression differences are above or below a threshold of significance.

When we combined classification results over all of the data sets (as in Figure 2), the PCA metric proved more sensitive than the other metrics. In this aggregate ROC analysis, the *Z *score performed second best, slightly outperforming the Wilcoxon *Z *score metric, which in turn slightly outperformed the KS metric. The sensitivity of the signed hypergeometric metric, which is arguably the most commonly applied method in gene expression analysis publications, was uniformly inferior to the other metrics and often not as sensitive as individual genes. The sensitivity of all methods declined as a function of decreasing pathway coherence, presumably because the activation signal from a coherent gene set, in which most of the genes are upregulated or downregulated in concert, is stronger than that from a set that is not coherent. The PCA activation metric is the least affected by this trend, retaining reasonable sensitivity even for incoherent gene sets (Figure 2d). Although PCA performed best in the combined classification test, it may not always be the best choice to use to interpret the biology of an expression data set. There were some data sets in which it did not perform as well as the other metrics (Supplemental Figures F3, F5, F6, F8 and F9 in Additional data file 1), but more importantly, because the principal component is highly data-set specific (that is, the weighting of individual genes is chosen to maximize the percentage of variance explained in that data set only), PCA may artifactually detect and use noise to discriminate between samples.

The number of pathways with significant changes in expression varied greatly across the nine data sets, and for some combinations of data sets and metrics no significant pathway expression changes were detected. For example, using PCA, two GEO data sets, GDS1062 [28] and GDS1221 [31], show no differentially activated gene sets at an estimated FDR of 0.2, suggesting that both sample subgroups are very similar to each other. Similarly, GDS1231 [32] shows only one activated gene set at the same FDR. The other six data sets showed large numbers of activated gene sets at all FDR levels. Finding no differentially activated gene sets for GDS1221, a study of response to the drug Gleevec (imatinib mesylate), is consistent with the findings of the original investigators [31]. Although the KS statistic performs better than chance and better than individual genes for this data set (p < 0.01; Supplemental Figure F3 in Additional data file 1 and Supplemental Table T3 in Additional data file 2), no activated pathways are found for an estimated FDR <0.2. O'Donnell *et al*. [28] used gene expression to classify non-metastatic versus metastatic head and neck cancer, deriving a 116-gene set of differentially expressed genes that correctly classified the training samples and a limited set of test samples. Their discussion does not identify any known biological gene sets that are consistently up- or downregulated between subgroups. Here, several pathway metrics, the *Z *score and Wilcoxon *Z *in particular, are able to do so (Supplemental Figure F9 in Additional data file 1, Supplemental Table T9 in Additional data file 2).

Not only does the detection sensitivity of the metrics vary (Figure 2), the pathways identified by them as differentially regulated are often different. To explore this quantitatively, we ranked the pathways by the statistical significance of their differences between each pair of sample groups using the *p *value from a two-sided Wilcoxon rank sum test for equal medians (see Materials and methods). This analysis shows that often a pathway that is detected as significant for one metric is not detected as significant by another. Motivated by these differences, we further compared the similarity of the five metrics by computing the Spearman (rank) correlation between them. Using all nine data sets we measured their correlation as a function of the FDR and coherence. At low FDRs we computed the Spearman correlation using only the most strongly differentially activated gene sets, while at higher FDRs we include a progressively larger subset of the 1,401 gene sets. Interestingly, we found the correlation between metrics depended only weakly on the FDR. Table 2 contains representative correlations for a FDR of 0.05 and coherence *p *value < 0.05. Although the exact correlation values vary with the FDR and coherence range, the Z-score, Wilcoxon, and KS metrics all had similar gene set rankings. However, the correlation of these three metrics to the PCA and hypergeometric metrics was substantially weaker. The correlation of these three with PCA was weaker still when incoherent sets were included (data not shown), indicating that pathway interpretations using different metrics are more consistent for coherent than for incoherent sets.

We can conclude from the above analyses that the list of pathways significantly changed between two sets of biological samples is strongly dependent on the type of data set (for example, the number of individual genes differentially expressed), the selected pathway activation metric, and whether or not 'incoherent' pathways are removed. For deeper exploration of these points, we use the data set of Farmer *et al*. [34], focusing on the differences between two estrogen-receptor (ER) negative subsets of breast cancer samples, termed 'basal' and 'apocrine'. Gene expression differences between breast cancer samples are dominated by ER status and so, as expected, the differences between basal and apocrine subtypes are relatively subtle. In fact, using the hypergeometric metric for gene-set activation, none of the 1,401 pathways are found to be differentially expressed at a FDR of 30%. The Wilcoxon and KS metrics are also relatively insensitive for this data set (Supplemental Figure F1 in Additional data file 1, Supplemental Table T1 in Additional data file 2). Neither of the metrics detects any activated pathways in the apocrine versus basal comparison with a FDR of 1%, and each detects only three pathways with a FDR of 10% (with one pathway in common). In contrast, many activated pathways are detected by the PCA metric, even at a FDR of 1%. As noted above, however, the sensitivity of the PCA metric may be spuriously high because the principal component adapts to the data set, so it is not clear all statistically significant pathways are biologically significant. At a FDR of 10%, for the total of the 71 gene sets with a coherence *p *value < 0.01, the *Z *score activation metric detects 22 activated gene sets and the PCA metric detects 41.

Of the 22 gene sets detected at a FDR of 10% by the *Z *score metric in the apocrine versus basal data set, almost all are related either to the cell cycle or to protein and amino acid metabolism. Compared to basal-type breast cancer samples, apocrine-type cancers demonstrate consistently lower activation of gene sets related to the cell cycle, particularly mitosis, and higher levels of activation of gene sets related to regulation of protein synthesis. The inflammatory response (presumably related to the infiltration of lymphocytes into the tumor) is lower in apocrine-type samples. If we rank pathways by the number of metrics showing a Wilcoxon rank sum *p *value for differential activation of <0.01, similar trends emerge; in addition, multiple sex-hormone related pathways demonstrate increased activation in apocrine-type cancers. This latter finding is consistent with the main hypothesis of Farmer *et al*. However, our conclusion that mitotic cell cycle pathways (for example, the pathways 'mitosis', 'nuclear division', 'spindle', 'cell cycle', 'mitotic cell cycle', 'regulation of mitosis') are expressed at significantly higher levels in apocrine samples relative to basal samples - detected by both *Z *score and PCA at a FDR of <10% - is not made by Farmer *et al*. This indicates the potential value of using multiple methods for assessing pathway activation. Likewise, several of the pathways listed in Farmer *et al*. as significantly upregulated in the apocrine samples (for example sulfur, lipid, and alcohol metabolism) do not pass the coherence threshold of *p *< 0.01. The expression of the individual genes in the sulfur metabolism pathway is shown in Supplemental Figure F11 (Additional data file 1) as one example. Although the expression of several individual genes in these pathways can separate the two tumor types, the vast majority of the genes in these pathways are not differentially expressed between the two sample types, and biological conclusions about the pathways' differential expression may not be warranted.

### Atlas of human gene expression

As a second illustration of the methods described above, we compiled a human gene expression atlas of approximately 11,000 RefSeq transcripts in 44 normal tissues and 8 cell lines. Most of the data were obtained by re-analysis of expression data from a genome-wide scan of alternative splicing as described in Materials and methods [35]. The data were previously available only at the probe level, but are now organized by transcript and gene. Five additional samples (pancreas, kidney, and three cell lines) were also re-hybridized for this study to improve data quality and coverage. Each normal tissue sample was made from a pool of individual donors. Finally, because the probes in this splicing study measured the expression of every exon-exon junction throughout each transcript, the median intensity of all probes for all transcripts representing a given gene provides a more robust measure of the gene's expression than array experiments using a single probe or set of probes near the 3' end of a single transcript. The expression data and their associated errors are provided in Supplemental Tables T10 and T11 (Additional data file 2).

### Human pathway expression map

**Figure 3:**
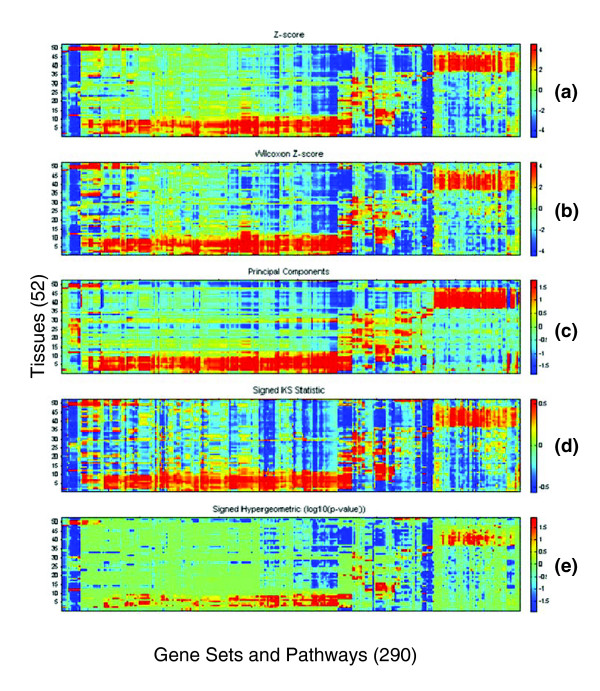
Comparison plot of human body atlas pathway expression computed by five different activations metrics: (a) *Z *score, (b) Wilcoxon *Z *score, (c) PCA, (d) signed KS, (e) signed hypergeometric. The rows are 52 tissues and cell lines (rows) and the columns are 290 gene sets and pathways. The order of pathways on both axes was determined by standard two-dimensional hierarchical clustering of the *Z *score results, and is the same as in Figure 4.

As described above, we first removed gene sets with incoherent expression over the samples in this study, resulting in 290 coherent gene sets (Supplemental Table T12 in Additional data file 2). The discarded sets and pathways may, of course, be actively transcribed and highly relevant in certain cell types within human tissues and yet represent only small fractions of the RNA populations within these tissues. For each coherent gene set in each tissue, we analyzed the expression level of that set using each of the five pathway activation metrics. Each resulting map is a matrix of 52 tissues and cell lines versus 290 gene sets and pathways (Figure 3). Results for the different metrics were more similar than for most of the experiments described above, possibly because of the larger differences in gene expression among body tissues. Although the maps in Figure 3 are broadly alike, specific differences in the maps are visible upon inspection. For example, the relative insensitivity of the signed hypergeometric metric is easily seen. Although the *Z *score did not perform as well as PCA in the combined ROC analysis described above, we selected it for further analysis and discussion of the human body atlas data because it characterizes each gene set with an intuitive interpretation as induced or repressed, provides a magnitude of activation that can be used in further analyses, had a similar pathway expression profile to PCA (Figure 3), and is less susceptible to fitting to noise in the data.

**Figure 4:**
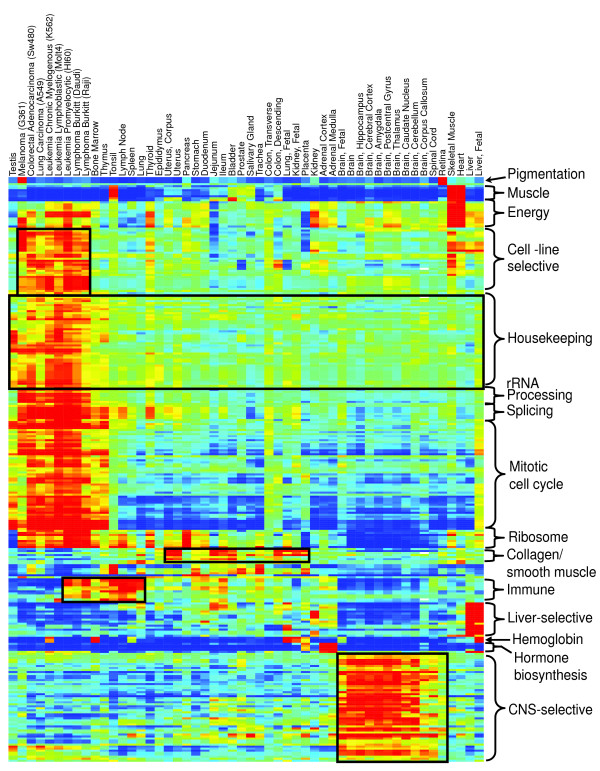
The tissue distribution of human gene pathways. A matrix of 52 tissues and cell lines (columns) versus 290 gene sets and pathways (rows). Each cell in the matrix indicates the *Z *score, the degree to which the genes in the pathway are over- or under-expressed relative to average (see Materials and methods). Both axes have been clustered with standard two-dimensional hierarchical clustering. A high resolution version of this figure with row labels and a table of expression *Z *scores of each set in each sample are available as supplemental materials from [21].

The *Z*-score map is shown at higher resolution in Figure 4. The expression patterns of the gene sets in the figure range from tissue-specific to ubiquitously expressed. At one extreme, the gene sets representing phototransduction, steroid hormone metabolism, and muscle filaments are expressed uniquely in retina, adrenal gland, and muscle, respectively. At the other extreme, sets expressed in all tissues in the atlas ('housekeeping' pathways) include those representing chromatin modification, RNA splicing, the ribosome, and mRNA processing. The largest set of tissue-specific pathways is unique to the brain. In what follows, references are made to a series of 'blocks' in Figure 4 that represent clusters of related gene sets with unique patterns of tissue expression. A higher resolution figure including all of the pathway names is provided as Supplemental Figure F12 (Additional data file 1), along with the gene sets in each block (Supplemental Table T12 in Additional data file 2), and the full table of Z-scores for every pathway in every tissue (Supplemental Table T13 in Additional data file 2). Specific gene set names are followed by CC, BP, or KG, according to whether the gene set was derived from the Gene Ontology Cellular Component hierarchy, the Gene Ontology Biological Process hierarchy, or KEGG pathways, respectively.

The pigmentation block consists of gene sets related to melanin synthesis, expressed at high levels in retina and a melanoma cell line. The eight gene sets in the muscle block are specifically expressed in heart and skeletal muscle, and includes expected categories such as 'sarcomere' (CC), 'myofibril' (CC), and 'regulation of muscle contraction' (BP). Two sets ('muscle contraction' (BP) and 'muscle development' (BP)) are also active in smooth muscle. Interestingly, these gene sets are also expressed in the tonsil sample; this is assumed to be a contaminant from the dissection process. This contamination was much easier to identify by upregulation of a muscle-specific pathway as a whole than by inspection of individual genes, illustrating the utility of the pathway expression map for quality control of tissue samples.

The energy block consists of gene sets of mitochondrial proteins, most highly expressed in striated muscle and at moderate levels in cancer cell lines, thyroid, and kidney. Activation of these energy pathways is not observed in some normal tissues with high expression of cell cycle-related gene sets, such as testis, bone marrow, and thymus. This shows that high cell turnover does not necessarily imply high levels of energy utilization. Examination of the expression of component genes from a representative pathway from this block, 'oxidative phosphorylation' (BP), demonstrates that there is coherent activation of approximately two-thirds of these genes in skeletal muscle, heart, and cell lines, accounting for the strong activation in these tissues, with only scattered activation of other genes in this GO category in other tissues (Supplemental Figure F13 in Additional data file 1). This coherently activated set of genes consists primarily of mitochondrial ATPases, and most of the apparent activation in other normal tissues, including brain, is accounted for by lysosomal (vacuolar) ATPases. The expression in thyroid is presumably related to the fact that lysosome formation is part of the pathway for cleavage of active thyroid hormone from thyroglobulin for release into the circulation. In kidney, vacuolar ATPases are essential for bicarbonate resorption in the nephron [36]. These observations highlight the potential for further improvement in these gene sets by refining their membership or dividing them into smaller groups, as we discuss in more detail below.

The cell-line selective block includes tRNA metabolism and proteasome subunit gene sets, indicating that certain aspects of protein biosynthesis and degradation are highly and selectively activated in malignant cells but not in normal highly proliferative tissues like bone marrow, thymus and testis. The differential expression of proteasomal genes seen here may be partly related to the increased susceptibility of cancer cells versus normal cells to proteasome inhibitors like bortezomib [37]. The housekeeping block comprises 55 gene sets expressed at high levels in cell lines and proliferating normal tissues, but expressed at intermediate levels in all tissues. These consist primarily of pathways related to gene transcription, messenger RNA processing and splicing, and nuclear export/import. The mitotic cell cycle block is a collection of gene sets strongly upregulated in cell lines relative to normal tissues, and expressed at moderate levels in bone marrow, testis, thymus, gut, and fetal brain and liver. The majority of these pathways are related to DNA synthesis or repair and to regulation of the cell cycle. In all cases, the activity of these pathways is higher in fetal tissues than in the corresponding adult tissues [38]. Pathways in the ribosome block consist largely of ribosomal proteins and show a broader distribution of expression across tissues than the pathways in the ribosomal rRNA-processing block discussed above. They are strongly expressed in the rapidly dividing tissues such as the cell lines and also expressed in tissues in which protein synthesis for export is active (pancreas, thyroid, and lymph nodes). The ribosome gene sets are expressed at very low levels in testis and subregions of adult brain. Lower expression of the ribosome in non-proliferative tissues is expected, and the similarity of ribosomal expression in brain and testis has been previously reported [39].

The collagen/smooth muscle block consists of six pathways relating to smooth muscle contraction or collagen production. As expected, these gene sets are expressed primarily in mesenchymal tissues and not expressed in brain or cell lines. The immune block consists of gene sets specific to lymphoid-derived tissues (antigen presentation and processing, B- and T-cell activation). These gene sets are expressed at high levels in lymphoid tissues and the two lymphoma cell lines studied here. These gene sets are also expressed in other tissues, particularly gut, probably representing the normal presence of lymphocytes in gastrointestinal tissue in the form of Peyer's patches.

The liver-selective block contains five sub-blocks of gene sets, all of which are highly upregulated in liver and fetal liver. Some of these sub-blocks also appear to be upregulated in other tissues. For example, while the complement pathway is strongly activated in liver and fetal liver, some activity is also seen in gut and lung. Recent reports support the existence of locally, that is, extrahepatically, synthesized complement [40]. Acute phase response activation in fetal lung may similarly be related to inflammation, while the apparent activation of lipid transport may be related to surfactant synthesis. The hemoglobin block is made up of genes for various hemoglobins, serving as markers for hematopoiesis. Tissues with high gene set expression levels include fetal, but not adult, liver and kidney, and lung and bone marrow. Expression was also noted in placenta. Expression of hemoglobin genes in the erythroleukemia line K562 (Supplemental Figure F14 in Additional data file 1), observed here, has been described previously [41]. Expression in fetal liver reflects the fact that the liver is a primary location for hematopoiesis in the fetus; we are unable to explain the apparent expression of these genes in fetal kidney and lung.

The hormone biosynthesis block contains genes involved in sterol biosynthesis (adrenal tissue and liver), which includes cholesterol synthesis, and more specific pathways relating to the synthesis of C-21 steroids, such as progesterone, glucocorticoids, and mineralocorticoids (adrenal tissue and placenta). Finally, the CNS-selective block consists of a series of pathways that are largely specific to neural tissue, including corpus callosum, spinal cord, retina, and brain. These pathways cover a multitude of aspects of nerve cell growth and signaling, including nerve maturation, axonic transport and ion channels, glial cell growth and differentiation, synaptic transmission, neurotransmitter regulation, perception of pain, as well as gene sets for Alzheimer's and Parkinson's diseases. Some of the apparent expression of these pathways in other tissues arises from the properties of the GO hierarchy. For example, the Biological Process classification 'Sodium ion transport' includes both genes expressed in neurons and genes expressed in colon and renal tubules, while the 'Microtubule-based process' classification and related gene sets includes genes involved in mitosis (and thus highly expressed in cell lines) and in axoplasmic transport (and thus highly expressed in neural tissue). Sodium and potassium transport pathways are also expressed in gut.

### Non-uniform expression of pathways and gene sets

**Figure 5:**
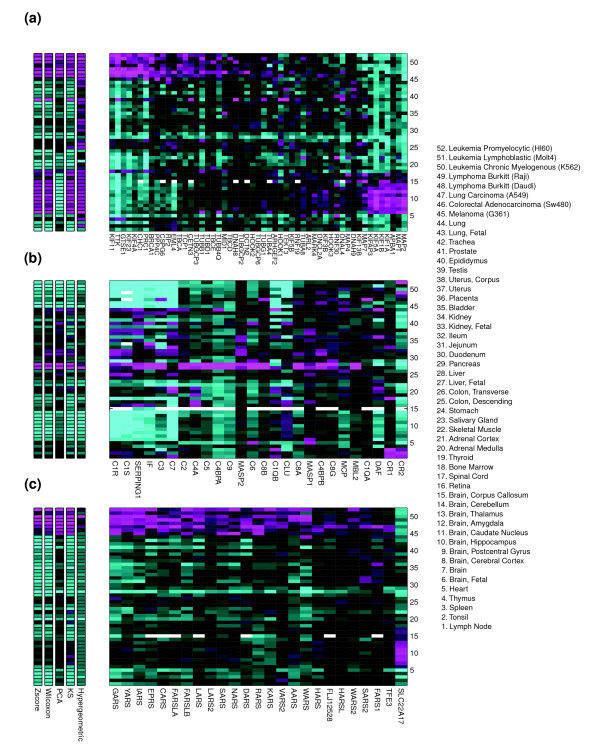
Expression of component genes for three gene sets over the tissues in the expression atlas showing varying patterns of expression coherence among the component genes. Shown to the left of each gene set are the pathway measurements calculated using each of the five activation metrics. Expression data are log10 ratio relative to average (see Materials and methods). Magenta and cyan indicate higher and lower expression of a gene or pathway in a given sample, respectively. The x-axis of each plot lists the component genes for each of three pathways: **(a) **'Microtubule-based process'; **(b) **'Complement Activation, Classical Pathway'; and **(c) **'tRNA aminoacylation'. All are GO Biological Process categories. The 52 tissues and cell lines used in this study are listed on the y-axes of each plot. Color axes are from -0.75 to 0.75 for gene expression log10 ratios (right plots). Missing data points are in white. For the activation metrics the color axes are normalized by the maximum and minimum values to range from 0 to 1.

The gene sets used in the pathway map above have relatively consistent expression of their constituent genes because we have filtered out the sets with the least coherent expression over the 52 human mRNA samples. In most of the gene sets that remain there is a large group of regulated genes and a smaller number of discordantly regulated genes. In many cases, however, a gene set passing the coherence filter still contains one or more genes with markedly different expression pattern from the global pattern. The five pathway activation metrics sometimes treat these cases differently. Three examples of this are discussed below and shown in Figure 5.

The GO Biological Process gene set 'Microtubule-based process' is composed largely of tubulins and kinesins and provides the first example. This gene set contains genes with two major patterns of expression (Figure 5a). The first subset is highly expressed in proliferative tissues, such as cell lines, testis, bone marrow, and colon, and contains mitotic kinesins like *KIF11*. A second major subset of genes is expressed at low levels in the proliferative tissues, but at high levels in neural tissues. This set contains genes involved in organelle transport, synaptic transmission, and synaptogenesis, like *KIF1A*, *KIF5A*, and *MAP2*. The rest of the genes display little correlated expression with either of the two major subsets or with each other. All of the pathway metrics consider this gene set activated, but for many samples PCA and hypergeometric disagree with the consensus of other metrics on the sign of the activation (Figure 5a, left panel). Because the two major sets have complementary tissue expression, for most set-based analyses it is more useful to consider them as biologically distinct processes - and appropriately there are GO Biological Process terms to represent these sub-processes. This example suggests how gene set analyses of gene expression may offer an important mechanism for dividing gene sets into biologically meaningful subsets with more specific annotations. Automated methods could be developed, for example to subdivide pathways in high-throughput by clustering the expression patterns of component genes.

The GO Biological Process category 'Complement Activation, Classical Pathway' illustrates another kind of complexity that may occur (Figure 5b). Here, one connected biological process is made up of proteins that reside in different tissues, so coherent expression over this data set is not expected. For example, most of the pathway components are enriched in liver, where complement proteins are produced. However, transcripts coding for complement components C1R and C2R are also observed in tissues where antigens are encountered (for example, intestines and lung, but not brain, testis or bone marrow), and the messenger RNAs for inflammatory response mediators such as complement receptors CR1 and CR2, which are expressed on lymphoid cells, are seen at higher levels in spleen, tonsil, and lymph node.

In a third example, the gene set representing 'tRNA aminoacylation', one gene (*SLC22A17*) deviates from the dominant expression pattern of the set, which shows higher expression in proliferative tissues, and instead shows high expression in brain (Figure 5c). In this case, the gene's membership in the tRNA aminoacylation GO Biological Process is somewhat incongruous with its annotation as a brain-specific organic ion transporter. Both of these annotations, tRNA aminoacylation and organic ion transport, are derived from automated sequence similarity searches using the Interpro database [42]. Deeper investigation of the sequence homology reveals that the Interpro hit for 'tRNA ligase' (IPR001412) occurs within the sequence matching the transporter Interpro domain (IPR007114, 'Major Facilitator Superfamily') and is a lower confidence match, suggesting it is spurious, and the gene does not play a role in tRNA aminoacylation. Of the five pathway metrics, KS seems to be most affected by the outlier, but all of the metrics appear to capture the dominant signal correctly. This example also shows how analysis of gene set expression can be effective in identifying possible annotation errors, and serves as a reminder that annotations of gene set membership, particularly those derived from automated procedures, should not be taken as fact.

Although there are set-specific differences in how the five metrics handle incoherent expression within these three gene sets, overall, the examples highlight the strong correlation of the Wilcoxon, KS, and *Z *score metrics, and the divergence of the PCA and hypergeometric metrics from the others. It is also clear from these examples and others in our analysis that few gene sets and pathways in public databases comprise single molecular machines whose components are always co-expressed. Two conclusions can be drawn from this. First, although analysis of pathways and gene sets is a powerful way to simplify and expedite analysis of genomic data sets, caution is necessary because of the immaturity of the current set annotations and the complexity of the underlying biology, and refinement of many of the sets will be required. Second, systematic examination of the expression-coherence of component genes over a data set (like the human expression atlas here) is a promising approach for refinement and extension of gene set and pathway membership. It should also be noted that different coherence filters may apply to each data set or experimental regime. Pathways perturbed by drug treatment or a disease state, for instance, could differ from those observed in a large set of pooled, normal tissues as presented here.

### Methods of measuring pathway expression

Arguably the most common method for analyzing experimental gene lists for pathway enrichment involves setting a threshold for differential expression of genes, and then using the hypergeometric distribution to determine if there is an excess number of induced or repressed genes in a predefined pathway relative to what is expected by chance [8,19,43]. Another reported method, called 'gene set enrichment analysis' (GSEA) analyzes a ranked list of all genes on a microarray (not just those differentially expressed above a threshold) for statistical enrichment of membership in gene sets and pathways among the top-ranking genes [16]. In subsequent work [44,45], a more computationally intensive version of GSEA was introduced. The KS activation metric used in this study is very similar to the original implementation of GSEA, but is less computationally intensive. Kim and colleagues [23] introduced PAGE (parametric analysis of gene set enrichment), which is similar to the Z-score metric, and compare it (favorably) to GSEA. Principal component analysis has been used in previous studies as well [46,47]. In a recent review of microarray analysis methods, Curtis *et al*. [48] compared the number of significant gene sets found by GSEA with two metrics based on the hypergeometric distribution and concluded that more downregulated pathways were found by GSEA.

In our analysis, the PCA metric was the most sensitive at discriminating between two subgroups across multiple data sets and the least affected by incoherent gene sets. The Z-score, Wilcoxon *Z *score, and KS metrics had approximately equal results in the ROC comparison and showed a high correlation of detected pathways (Table 2). The signed hypergeometric metric was inferior to all of the others. The sensitivity of all methods declined as the coherence of the gene sets they were measuring declined. PCA is also useful in providing an estimate for the coherence of a gene set over a given set of experiments, and was used here as our coherence filter (see Materials and methods). In spite of the superior performance of the PCA metric in class discrimination, it has two limitations: it is data-set dependent and its output is not readily interpretable. In contrast, the *Z *score is simple in interpretation, capturing both the magnitude and significance of expression of the gene set and, in the case of ratio experimental data, the direction of change. Another potential advantage of a collective activation score like the *Z *score is that it lends itself easily to analysis techniques such as clustering, correlation, or analysis of variance, while *p *values from enrichment calculations do not. However, the *Z*-score has limitations as well. It is somewhat sensitive to outliers and in some cases induced and repressed genes from the same pathway may cancel each other out and prevent detection.

## Conclusion

There are many advantages to performing analysis of microarray data at the gene set and pathway level. First, a global view of the behavior of defined biological modules is more intuitive than expression levels of hundreds or thousands of individual genes, and biological interpretation is much faster than analyzing a list of genes with significant expression changes because functionally understood gene sets with significant expression changes are identified automatically. Gene set analysis can also provide a better signal-to-noise ratio than the analysis of individual genes, and can detect coordinated activation of a pathway whose components would not pass single-gene significance thresholds. Finally, analysis techniques normally used at the gene-level to analyze individual genes such as correlation, clustering, and analysis of variance may be leveraged to analyze gene sets and offer additional insights. The gene-level expression atlas will also be useful for identifying new functional groups of genes or gene interaction networks through analysis of co-expressed gene clusters, as previous reports have shown [47,49-53]. We have illustrated the general utility of this approach by constructing a map of the expression of human pathways and gene sets over a large, robust set of gene-level expression data. This map is consistent with known tissue-specific pathways and provides new insights into the tissue distribution of other pathways and processes.

We also showed by comparing the sensitivities of five measures of pathway expression over nine other mRNA expression data sets that conclusions about pathway activation are sensitive to the method of measurement and whether or not incoherent pathways are excluded. The most appropriate pathway analysis is, therefore, dependent on the data set being analyzed, since the subset of coherent pathways is highly variable over different experiments. We recommend using coherence filtering in all cases to select the pathways most relevant to the samples in the study. When there are large gene expression differences between samples, all of the methods performed well, with mostly similar results. In this situation, we recommend using the *Z *score metric or one of the non-parametric methods (KS, Wilcoxon) and avoiding the over-fitting to a specific data set that can occur with PCA. For more subtle perturbations with limited numbers of genes differentially regulated, PCA was clearly the most sensitive and hypergeometric the least. To achieve maximum biological sensitivity and accuracy in these cases, we recommend using PCA, combined with at least one of the other three methods, *Z *score, KS, or Wilcoxon *Z*.

## Materials and methods

### DNA microarray data

The nine data sets used to compare the gene-set activation metrics were selected from eight studies in the GEO database. Each data set contained two relatively homogeneous subsets of samples. One study (GDS1329) provided two data sets. These subsets consisted of a baseline type and pathological samples or, in some cases, two different but related disease types. (Samples not in either subset were omitted from the comparisons.) We treated these single-channel data sets as ratio data sets by computing the median for each gene over all the baseline samples and dividing all expression values by the corresponding median and taking the base-10 logarithm. For each data set, Table 1 contains the GEO identifier and nature and sizes (in parentheses) of the two sample subgroups. In each data set the samples in Subgroup 1 constitute the baseline set.

To create the human body atlas, oligonucleotide probes were placed at each exon-exon junction of 11,138 RefSeq transcripts [35]. Purchased mRNA from 44 tissues in normal physiological state, pooled from multiple individuals, and 8 cell lines were amplified and labeled using a full-length amplification protocol and hybridized in duplicate in a two-color dye swap experiment[54]. In Johnson *et al*. [35], six of 52 tissues contained data for only 80% of the genes. For five of these tissues (pancreas, kidney, Burkitt's lymphoma (Raji), lung carcinoma (A549), and melanoma (G361)), new hybridizations were performed here to fill in the missing data. After background normalization, the intensity value of each probe in each tissue was divided by the average intensity across all 52 tissues to determine a ratio, and then the log10 of that ratio used for further analysis. Standard deviations (SDs) for each intensity measurement were calculated using the equation:

SD=a+b∗intensity
 MathType@MTEF@5@5@+=feaafiart1ev1aaatCvAUfeBSjuyZL2yd9gzLbvyNv2Caerbhv2BYDwAHbqedmvETj2BSbqee0evGueE0jxyaibaiKI8=vI8tuQ8FMI8Gi=hEeeu0xXdbba9frFj0=OqFfea0dXdd9vqai=hGuQ8kuc9pgc9s8qqaq=dirpe0xb9q8qiLsFr0=vr0=vr0dc8meaabaqaciGacaGaaeqabaqadeqadaaakeaacaqGtbGaaeiraiabg2da9maakaaabaGaamyyaiabgUcaRiaadkgacqGHxiIkieGacaWFPbGaa8NBaiaa=rhacaWFLbGaa8NBaiaa=nhacaWFPbGaa8hDaiaa=LhaaSqabaaaaa@4204@

where *a *= 100 and *b *= 0.2 were empirically derived from individual same-versus-same and same-versus-different hybridization experiments and represent single-hybridization, single-probe estimates of background (*a*) and fractional error (*b*). As we used multiple probes per gene and two hybridizations per sample pair (a dye-swap), final error estimates for gene expression are a combination of both propagation of this model measurement error and variance over the repeat measurements. These error estimates were then propagated to ratio and log10 ratio error estimates (Supplemental Tables T10 and T11 in Additional data file 2). Since the initial array design, NCBI has removed over 300 of the RefSeq transcripts from their databases. After removing these transcripts and any other transcripts currently unmapped to Entrez gene identifiers from our data set, the remaining 10,815 RefSeq transcripts map to 9,982 genes. Finally, using all gene-associated probes, we calculated an error-weighted average of log10 ratios for each gene in each tissue. Probe-level expression data have been deposited in the GEO database [35] (GSE740), and all gene and pathway expression data are available online [21].

### Gene sets and coherence filtering

We compiled 1,281 gene sets from the 1 November 2004 Release of GO (241 from cellular component and 1,040 from biological process), and 117 gene sets from KEGG Release 33, downloaded 11 January 2005. The mean number of genes in each set was 23.8 ± 28.5 (mean ± SD; minimum 1, maximum 159). To build the human pathway expression map we reduced these to 290 gene sets (23 from KEGG, 89 from the GO Cellular Component hierarchy, and 178 from the GO Biological Process hierarchy) by applying two filters. First, we required that each gene set retained contain at least five genes and no more than 200 genes. Second, we filtered gene sets based on their coherence, the percentage of total variance of the expression values within a gene set captured by the first principal component across all tissues. This idea has been discussed previously [20], although we used a different test for coherence here. To determine the appropriate cutoff for a gene set of size |*S*|, we generated 1,000 random gene sets of size |*S*|, and calculated the distribution of coherence values. The random-set coherence distribution was approximately normal, although its mean and standard deviation were size-dependent. Of the initial 1,401 gene sets, 290 had a coherence over the human body atlas data set that was more than 2.6 standard deviations greater than the mean of the random-set distribution for that size (corresponding to a one-sided p value of 0.005), and these 290 sets were retained for further analysis. The mean number of genes in these 290 coherent gene sets was 33.8 ± 32.9 (mean ± SD; minimum 5, maximum 159).

Some of the 290 gene sets overlap in component genes, and some gene sets are subsets of others. This is due to the hierarchical nature of GO and functional overlap with gene sets in KEGG. Rather than merge these sets we kept them all in order to maximize the functional annotation conveyed by the gene set names. To measure the overlap between two gene sets we used the average of the two ratios of the number of genes in the intersection of the two gene sets to the total number of genes in each gene set. The overlap is most significant between gene sets in the same block ranging from a low of 7% in the Cell-selective block to a high of 85% in the Hemoglobin block with a mean within-block overlap over all 14 blocks of 31%.

### Measuring gene set expression

We compared five gene-set activation metrics. Given a gene *g*, let *X*_*tg *_be the expression value (log10 fold change, relative to background) for gene *g *in tissue *t*. Let *S *be the set of genes in a pathway. For tissue *t*, if <*X*_*tS *_> and <*X*_*t *_> are the mean of *X*_*tg *_over the genes in *S *and all the genes on the microarray, respectively, and σ_*t *_is the standard deviation of *X*_*tg *_over all the genes on the microarray, then the Z-score activation metric used to measure the relative expression level of pathway *S *in tissue *t *is:

ZtS=<XtS>−<Xt>σt|S|
 MathType@MTEF@5@5@+=feaafiart1ev1aaatCvAUfeBSjuyZL2yd9gzLbvyNv2Caerbhv2BYDwAHbqedmvETj2BSbqee0evGueE0jxyaibaiKI8=vI8tuQ8FMI8Gi=hEeeu0xXdbba9frFj0=OqFfea0dXdd9vqai=hGuQ8kuc9pgc9s8qqaq=dirpe0xb9q8qiLsFr0=vr0=vr0dc8meaabaqaciGacaGaaeqabaqadeqadaaakeaacaWGAbWaaSbaaSqaaiaadshacaWGtbaabeaakiabg2da9maalaaabaGaeyipaWJaamiwamaaBaaaleaacaWG0bGaam4uaaqabaGccqGH+aGpcqGHsislcqGH8aapcaWGybWaaSbaaSqaaiaadshaaeqaaOGaeyOpa4dabaGaeq4Wdm3aaSbaaSqaaiaadshaaeqaaaaakmaakaaabaWaaqWaaeaacaWGtbaacaGLhWUaayjcSdaaleqaaaaa@4825@

where |*S*| is the number of genes in *S*. The value of *Z *is expressed in units of standard deviation and is a measure of violation of the null hypothesis that the genes in *S *are independently sampled from a distribution similar to that of all the genes on the microarray. If the null hypothesis is valid, then *Z *will have approximately a standard normal distribution, and so a large positive value of *Z*_*t *_suggests collective upregulation of the genes in *S *(which we consider to represent 'activation' of *S*) in tissue *t*; a large negative value suggests collective downregulation. The normalization by |S|
 MathType@MTEF@5@5@+=feaafiart1ev1aaatCvAUfeBSjuyZL2yd9gzLbvyNv2Caerbhv2BYDwAHbqedmvETj2BSbqee0evGueE0jxyaibaiKI8=vI8tuQ8FMI8Gi=hEeeu0xXdbba9frFj0=OqFfea0dXdd9vqai=hGuQ8kuc9pgc9s8qqaq=dirpe0xb9q8qiLsFr0=vr0=vr0dc8meaabaqaciGacaGaaeqabaqadeqadaaakeaadaGcaaqaamaaemaabaGaam4uaaGaay5bSlaawIa7aaWcbeaaaaa@3742@ makes comparison of different-sized gene sets possible and reflects the fact that, for larger gene sets, even a slight collective shift in fold change can be significant.

Because the *Z*-statistic essentially measures a shift in location (mean expression) for the genes in *S*, we compared its sensitivity to several other possible signed measures of location shift, which were created by modifying, where necessary, standard statistics with a sign to indicate the direction of expression change. The Wilcoxon *Z *statistic is a well-known statistic that is calculated according to a similar formula, but using the ranks of the *X*_*tg *_among all genes in tissue *t*, rather than the actual fold changes. To calculate a signed KS statistic, we computed each of the two one-sided KS statistics, comparing the distribution of the expression values in *S *with the distribution of the genes on the microarray as a whole, and took the larger of the two statistics, with the appropriate sign. To calculate a hypergeometric *p *value, we used a threshold of two-fold differential expression (other threshold values showed qualitatively similar results, data not shown) to define an induced or repressed gene, and then calculated the probability that the relative enrichment of differentially expressed genes observed in a gene set in a particular tissue could have been observed by chance, using the hypergeometric distribution. To provide a sign for the hypergeometric *p *value, the calculation was done separately for the induced and repressed genes in each set, and the smaller of the two *p *value was used, as well as its 'sign' (negative if repressed genes were more enriched in the gene set than induced genes, positive otherwise). The relative insensitivity of the HG metric was little changed by varying the differential expression threshold. Finally, for the PCA statistic, we calculated *PC*_1_, the first principal component of the expression values of the genes in *S *across all tissues, and used the projection (scalar product) of the expression values in a tissue with *PC*_1 _as a measure of activation of the gene set in that tissue.

### ROC comparison of activation metrics

We compared the five activation metrics for measuring gene set expression, and the individual genes in the expression data set, for their detection sensitivity. We applied each metric to measure the activation of the gene sets that met a coherence threshold (*p *≤ 0.01, 0.05, 0.10, and 1.0) in each of the nine GEO data sets. For each data set we compared two classes that were known to be different (typically one class was normal and the other pathological). Each gene set was measured in each sample in each of the two classes by each metric. We used a two-sided Wilcoxon rank sum test for equal medians to test the null hypothesis that the activation metric values for each gene set in the two classes come from distributions with equal medians. The result of this test is quantified by the returned *p *value. The smaller the *p *value, the more unlikely is the null hypothesis that the gene set median values are equal. We performed this test between the two classes for all gene sets. In a similar manner, we used the same test to compare individual gene expression values between the two classes. We used the *p *value from the two-sided Wilcoxon rank sum statistic to compute a false detection rate for each p value threshold using the adaptive method of Benjamini and Hochberg [55] and displayed the results using ROC curves [56]. The x-axis is the proportion of false positives; the percent of gene sets that did not distinguish the two classes at the specified p value threshold. The y-axis is the true positive rate; the percent of gene sets that did distinguish the two classes at the specified threshold. The interval of [0, 0.3] was chosen to correspond to what might be an acceptable FDR. The percent of true positives varies between data sets and is presumably indicative of the type(s) of biological differences between the two classes in each data set.

## Additional data files

The following additional data are available with the online version of this paper. Additional data file1 contains supplemental figures. Additional data file2 contains supplemental tables.

## Supplementary Material

Additional data file 1 Supplemental figures Click here for file

Additional data file 2 Supplemental tables Click here for file

## Figures and Tables

**Table 1:** GEO identifiers and data sets used for pathway activation method comparison

GEO ID	Subgroup 1 (Baseline)	Subgroup 2
GDS1062	Metastasis-negative squamous cell carcinoma (8)	Metastasis-positive squamous cell carcinoma (14)
GDS1067	Monoclonal gammopathy (7)	Multiple myeloma (39)
GDS1210	Normal gastric tissue (8)	Gastric carcinoma (14)
GDS1220	Normal pleural tissue (8)	Malignant mesothelioma (40)
GDS1221	Peripheral blood, CML responsive Gleevec (9)	Peripheral blood, CML not responsive Gleevec (7)
GDS1231	Enriched for hematopoietic stem cells (9)	Enriched for committed hematopoietic cells (9)
GDS1329	'Basal' breast tumors (16)	'Luminal' breast tumors (27)
GDS1329	'Basal' breast tumors (16)	'Apocrine' breast tumors (6)
GDS1332	Whole blood normal controls (14)	Whole blood symptomatic Huntington disease (12)

The numbers in parentheses are the number of samples in each subgroup. CML, chronic myelogenous leukemia. References are provided in the main text.

**Table 2:** Spearman correlation of pathway activation metrics over the nine data sets of Table 1

	*Z*	HG	PCA	WC	KS
Z	1.0	0.30	0.53	0.77	0.64
HG		1.0	0.32	0.18	0.12
PCA			1.0	0.32	0.24
WC				1.0	0.85
KS					1.0

Spearman correlation of pathway activation metrics over the nine data sets of Table 1, with consistent FDR of 0.05 and coherence *p* value ≤ 0.05. Columns and rows are *Z *score (*Z*), signed hypergeometric (HG), principal component analysis (PCA), Wilcoxon *Z *score (WC) and Kolmogorov-Smirnov (KS).
