# Supplementary material for: Pathway and gene-set activation measurement from mRNA expression data: the tissue distribution of human pathways
Source: Genome Biol. 2006 Oct 17;7(10):R93. doi: 10.1186/gb-2006-7-10-r93 (PMC1794557; doi:10.1186/gb-2006-7-10-r93)
Supplement: Additional data file 1 — Supplemental figures [file gb-2006-7-10-r93-S1.doc]

# SUPPLEMENTAL FIGURES

# Pathway and gene-set activation measurement from mRNA expression data: the tissue distribution of human pathways

D. M. Levine et al.

**Supplemental Figure F1**
ROC analysis performed as in Figure 2 for the comparison of 16 basal breast cancer tumors with 6 apocrine breast cancer tumors (GEO database identifier GDS1329). Input pathways were filtered for coherence over this data set using a 0.01 p-value threshold.

**Supplemental Figure F2**
ROC analysis performed as in Figure 2 for the comparison of 16 basal breast cancer tumors with 27 luminal breast cancer tumors (GDS1329). Input pathways were filtered for coherence over this data set using a 0.01 p-value threshold.

**Supplemental Figure F3**
ROC analysis performed as in Figure 2 for the comparison of peripheral blood samples from 9 patients with CML responsive to Gleevec to samples from 7 patients with CML not responsive to Gleevec (GDS1221). Input pathways were filtered for coherence over this data set using a 0.01 p-value threshold.

**Supplemental Figure F4**
ROC analysis performed as in Figure 2 for the comparison of 8 normal gastric tissue samples with 14 gastric carcinoma samples (GDS1210). Input pathways were filtered for coherence over this data set using a 0.01 p-value threshold.

**Supplemental Figure F5**
ROC analysis performed as in Figure 2 for the comparison of 9 samples enriched for hematopoietic stem cells with 9 samples enriched for committed hematopoietic cells (GDS1231). Input pathways were filtered for coherence over this data set using a 0.01 p-value threshold.

**Supplemental Figure F6**
ROC analysis performed as in Figure 2 for the comparison of 14 samples of normal whole blood and 12 samples of whole blood symptomatic of Huntington disease (GDS1332). Input pathways were filtered for coherence over this data set using a 0.01 p-value threshold.

**Supplemental Figure F7**
ROC analysis performed as in Figure 2 for the comparison of 8 normal pleural tissue samples and 40 malignant mesothelioma samples (GDS1220). Input pathways were filtered for coherence over this data set using a 0.01 p-value threshold.

**Supplemental Figure F8**
ROC analysis performed as in Figure 2 for the comparison of 7 monoclonal gammopathy samples with 39 multiple myeloma samples (GDS1067). Input pathways were filtered for coherence over this data set using a 0.01 p-value threshold.

**Supplemental Figure F9**
ROC analysis performed as in Figure 2 for 8 metastasis-negative squamous cell samples with 14 metastasis-positive squamous cell samples (GDS1062). Input pathways were filtered for coherence over this data set using a 0.01 p-value threshold.

**Supplemental Figure F10**
ROC curve for human body atlas data performed as in Figure 2. Input pathways were filtered for coherence over this data set using a 0.01 p-value threshold.

**Supplemental Figure F11.** Heat map showing the expression values for the Sulfur Metabolism pathway.

**Supplemental Figure F12.** Matrix of 52 tissues and cell lines vs. 290 gene sets and pathways. Each cell in the matrix indicates the Z-score, the degree to which the genes in the gene set or pathway are over- or under-expressed relative to average. Bright red indicates significant overexpression relative to the average mRNA abundance in all tissues, and dark blue significant underexpression.


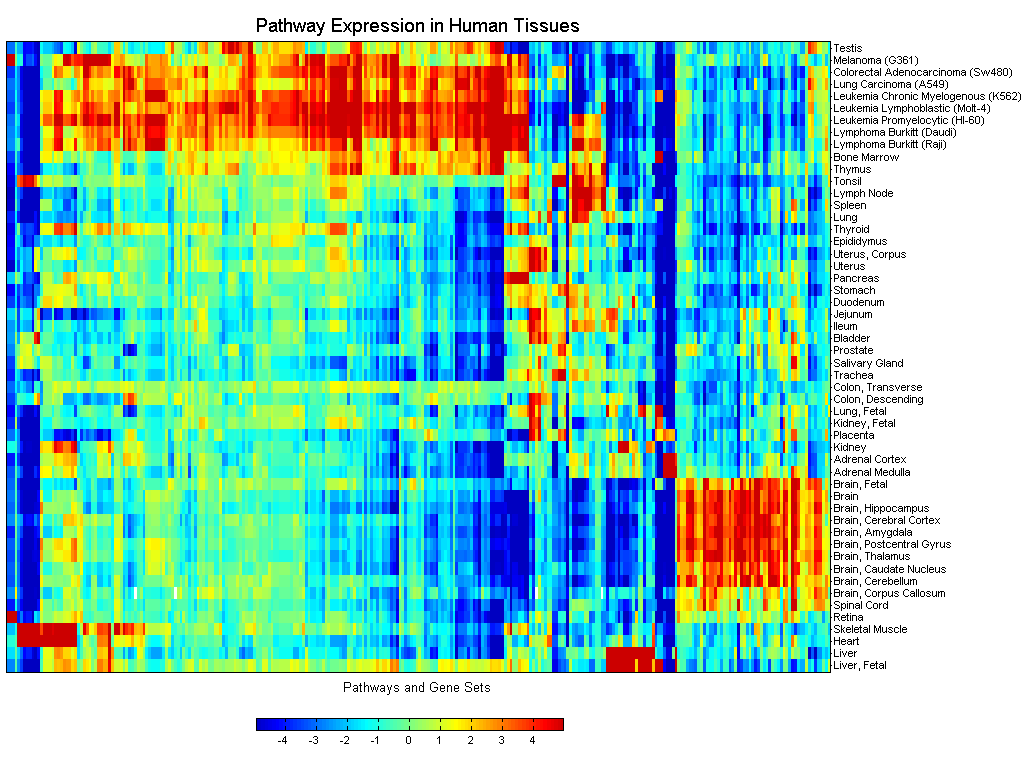


**Supplemental Figure F13.** Heat map showing the expression values for the component genes of the Oxidative Phosphorylation gene set.

**Supplemental Figure F14.**  Heat map showing the expression values for the component genes of the Hemoglobin gene set.
