# Supplementary material for: Pathway and gene-set activation measurement from mRNA expression data: the tissue distribution of human pathways
Source: Genome Biol. 2006 Oct 17;7(10):R93. doi: 10.1186/gb-2006-7-10-r93 (PMC1794557; doi:10.1186/gb-2006-7-10-r93)
Supplement: Additional data file 2 — Supplemental tables [file gb-2006-7-10-r93-S2.doc]

# SUPPLEMENTAL TABLES

# Pathway and gene-set activation measurement from mRNA expression data: the tissue distribution of human pathways

D. M. Levine et al.

**All of the files described below are provided online at** <http://pubinfo.rii.com/HumanPathwayExpression/index.html>.

**Supplemental Table T1**
Pathway activation metrics computed for every gene set or pathway in 16 Basal breast cancer tumors and 6 apocrine breast cancer tumors (GEO database identifier GDS1329). The file contains one header row and 7005 additional rows, one for each of the 1401 gene sets or pathways, each measured using five pathway activation metrics. The first column is the gene set or pathway name. The characters (KG), (BP), and (CC) at the end of the names correspond to the data source: Kegg, Gene Ontology Biological Process, and Gene Ontology Cellular Component, respectively. The second column contains the long name for the data source. The third column is the coherence p-value. The fourth column is the name of the pathway activation metric used to compare the two classes with a Wilcoxon rank sum test. The fifth column is the p-value from the Wilcoxon rank sum test. The next 16 columns contain the pathway activation metric values for each of the Basal breast cancer tumors. The last 6 columns contain the pathway activation metric values for each of the apocrine breast cancer tumors. Columns in the text file are delimited by a dollar sign ('$') character.

**Supplemental Table T2**
Pathway activation metrics computed for every gene set or pathway in 16 Basal breast cancer tumors and 27 Luminal breast cancer tumors (GDS1329). The file contains one header row and 7005 additional rows, one for each of the 1401 gene sets or pathways, each measured using five pathway activation metrics. The first column is the gene set or pathway name. The characters (KG), (BP), and (CC) at the end of the names correspond to the data source: Kegg, Gene Ontology Biological Process, and Gene Ontology Cellular Component, respectively. The second column contains the long name for the data source. The third column is the coherence p-value. The fourth column is the name of the pathway activation metric used to compare the two classes with a Wilcoxon rank sum test. The fifth column is the p-value from the Wilcoxon rank sum test. The next 16 columns contain the pathway activation metric values for each of the Basal breast cancer tumors. The last 27 columns contain the pathway activation metric values for each of the Luminal breast cancer tumors. Columns in the text file are delimited by a dollar sign ('$') character.

**Supplemental Table T3**
Pathway activation metrics computed for every gene set or pathway in 9 patients with CML responsive to Gleevec and 7 patients with CML not responsive to Gleevec (GDS1221). The file contains one header row and 6975 additional rows, one for each of the 1395 gene sets or pathways, each measured using five pathway activation metrics. The first column is the gene set or pathway name. The characters (KG), (BP), and (CC) at the end of the names correspond to the data source: Kegg, Gene Ontology Biological Process, and Gene Ontology Cellular Component, respectively. The second column contains the long name for the data source. The third column is the coherence p-value. The fourth column is the name of the pathway activation metric used to compare the two classes with a Wilcoxon rank sum test. The fifth column is the p-value from the Wilcoxon rank sum test. The next 9 columns contain the pathway activation metric values for each of the patients with CML responsive to Gleevec. The last 7 columns contain the pathway activation metric values for each of the patients with CML not responsive to Gleevec. Columns in the text file are delimited by a dollar sign ('$') character.

**Supplemental Table T4**
Pathway activation metrics computed for every gene set or pathway in 8 normal gastric tissue samples and 14 gastric tissue samples with carcinoma (GDS1210). The file contains one header row and 3640 additional rows, one for each of the 728 gene sets or pathways, each measured using five pathway activation metrics. The first column is the gene set or pathway name. The characters (KG), (BP), and (CC) at the end of the names correspond to the data source: Kegg, Gene Ontology Biological Process, and Gene Ontology Cellular Component, respectively. The second column contains the long name for the data source. The third column is the coherence p-value. The fourth column is the name of the pathway activation metric used to compare the two classes with a Wilcoxon rank sum test. The fifth column is the p-value from the Wilcoxon rank sum test. The next 8 columns contain the pathway activation metric values for each of the normal gastric tissue samples. The last 14 columns contain the pathway activation metric values for each of the gastric tissue samples with carcinoma. Columns in the text file are delimited by a dollar sign ('$') character.

**Supplemental Table T5**
Pathway activation metrics computed for every gene set or pathway in 9 samples enriched for hematopoietic stem cells and 9 samples enriched for committed hematopoietic cells (GDS1231). The file contains one header row and 6790 additional rows, one for each of the 1358 gene sets or pathways, each measured using five pathway activation metrics. The first column is the gene set or pathway name. The characters (KG), (BP), and (CC) at the end of the names correspond to the data source: Kegg, Gene Ontology Biological Process, and Gene Ontology Cellular Component, respectively. The second column contains the long name for the data source. The third column is the coherence p-value. The fourth column is the name of the pathway activation metric used to compare the two classes with a Wilcoxon rank sum test. The fifth column is the p-value from the Wilcoxon rank sum test. The next 9 columns contain the pathway activation metric values for each of the samples enriched for hematopoietic stem cells. The last 9 columns contain the pathway activation metric values for each of the samples enriched for committed hematopoietic cells. Columns in the text file are delimited by a dollar sign ('$') character.

**Supplemental Table T6**
Pathway activation metrics computed for every gene set or pathway in 14 samples of normal whole blood and 12 samples of whole blood symptomatic of Huntington disease (GDS1332). The file contains one header row and 6995 additional rows, one for each of the 1399 gene sets or pathways, each measured using five pathway activation metrics. The first column is the gene set or pathway name. The characters (KG), (BP), and (CC) at the end of the names correspond to the data source: Kegg, Gene Ontology Biological Process, and Gene Ontology Cellular Component, respectively. The second column contains the long name for the data source. The third column is the coherence p-value. The fourth column is the name of the pathway activation metric used to compare the two classes with a Wilcoxon rank sum test. The fifth column is the p-value from the Wilcoxon rank sum test. The next 14 columns contain the pathway activation metric values for each of the samples of normal whole blood. The last 12 columns contain the pathway activation metric values for each of the samples of whole blood symptomatic of Huntington disease. Columns in the text file are delimited by a dollar sign ('$') character.

**Supplemental Table T7**
Pathway activation metrics computed for every gene set or pathway in 8 normal pleural tissue samples and 40 malignant mesothelioma samples (GDS1220). The file contains one header row and 7005 additional rows, one for each of the 1401 gene sets or pathways, each measured using five pathway activation metrics. The first column is the gene set or pathway name. The characters (KG), (BP), and (CC) at the end of the names correspond to the data source: Kegg, Gene Ontology Biological Process, and Gene Ontology Cellular Component, respectively. The second column contains the long name for the data source. The third column is the coherence p-value. The fourth column is the name of the pathway activation metric used to compare the two classes with a Wilcoxon rank sum test. The fifth column is the p-value from the Wilcoxon rank sum test. The next 8 columns contain the pathway activation metric values for each of the normal pleural tissue samples. The last 40 columns contain the pathway activation metric values for each of the malignant mesothelioma samples. Columns in the text file are delimited by a dollar sign ('$') character.

**Supplemental Table T8**
Pathway activation metrics computed for every gene set or pathway in 7 monoclonal gammopathy samples and 39 multiple myeloma samples (GDS1067). The file contains one header row and 7005 additional rows, one for each of the 1401 gene sets or pathways, each measured using five pathway activation metrics. The first column is the gene set or pathway name. The characters (KG), (BP), and (CC) at the end of the names correspond to the data source: Kegg, Gene Ontology Biological Process, and Gene Ontology Cellular Component, respectively. The second column contains the long name for the data source. The third column is the coherence p-value. The fourth column is the name of the pathway activation metric used to compare the two classes with a Wilcoxon rank sum test. The fifth column is the p-value from the Wilcoxon rank sum test. The next 7 columns contain the pathway activation metric values for each of the monoclonal gammopathy samples. The last 39 columns contain the pathway activation metric values for each of the multiple myeloma samples. Columns in the text file are delimited by a dollar sign ('$') character.

**Supplemental Table T9**
Pathway activation metrics computed for every gene set or pathway in 8 metastasis-negative squamous cell samples and 14 metastasis-positive squamous cell samples (GDS1062). The file contains one header row and 7005 additional rows, one for each of the 1401 gene sets or pathways, each measured using five pathway activation metrics. The first column is the gene set or pathway name. The characters (KG), (BP), and (CC) at the end of the names correspond to the data source: Kegg, Gene Ontology Biological Process, and Gene Ontology Cellular Component, respectively. The second column contains the long name for the data source. The third column is the coherence p-value. The fourth column is the name of the pathway activation metric used to compare the two classes with a Wilcoxon rank sum test. The fifth column is the p-value from the Wilcoxon rank sum test. The next 8 columns contain the pathway activation metric values for each of the metastasis-negative squamous cell samples. The last 14 columns contain the pathway activation metric values for each of the metastasis-positive squamous cell samples. Columns in the text file are delimited by a dollar sign ('$') character.

**Supplemental Table T10**
Human body atlas data, GEO database identifier GSE740. Expression levels for every gene in every tissue. Each file contains one header row and 9,982 additional rows, one for each transcript. The first column is the gene symbol. The second column is the RefSeq transcript ID. Columns 3-54 correspond to the 52 tissues. Missing values are represented by the character string NaN. Columns in the text file are delimited by a dollar sign ('$') character.

**Supplemental Table T11**
Human body atlas data, GEO database identifier GSE740. Error estimates for every gene in every tissue. Each file contains one header row and 9,982 additional rows, one for each transcript. The first column is the gene symbol. The second column is the RefSeq transcript ID. Columns 3-54 correspond to the 52 tissues. Missing values are represented by the character string NaN. Columns in the text file are delimited by a dollar sign ('$') character.

**Supplemental Table T12**
Human body atlas data, GEO database identifier GSE740. Data for the 290 gene sets used for analysis. The first column is the block name from Figure 3 into which the gene set or pathway was classified. The second column is the gene set or pathway name. The third column is the source of the gene set or pathway, one of Kegg, Gene Ontology Biological Process, and Gene Ontology Cellular Component. The fourth column is the gene symbol. The fifth column is the RefSeq transcript ID. Columns in the text file are delimited by a dollar sign ('$') character.

**Supplemental Table T13**
Human body atlas data, GEO database identifier GSE740. The Z-score values computed for every coherent gene set or pathway in every tissue. Each file contains one header row and 290 additional rows, one for each gene set or pathway. The first column is the gene set or pathway name. The characters (KG), (BP), and (CC) at the end of the names correspond to the data source: Kegg, Gene Ontology Biological Process, and Gene Ontology Cellular Component, respectively. The second column contains just the long name for the data source. The third column is the coherence p-value. Columns 4-55 correspond to the 52 tissues in the same order as given in Figure 3. Columns in the text file are delimited by a dollar sign ('$') character.
